# Revealing the targets and mechanisms of vitamin A in the treatment of COVID-19

**DOI:** 10.18632/aging.103888

**Published:** 2020-08-15

**Authors:** Rong Li, Ka Wu, Yu Li, Xiao Liang, William Ka Fai Tse, Lu Yang, Keng Po Lai

**Affiliations:** 1 Guangxi Key Laboratory of Tumor Immunology and Microenvironmental Regulation, Guilin Medical University, Guilin, China; 2 Department of Pharmacy, The Second People's Hospital of Nanning City, The Third Affiliated Hospital of Guangxi Medical University, Nanning, China; 3 Center for Promotion of International Education and Research, Faculty of Agriculture, Kyushu University, Fukuoka, Japan

**Keywords:** vitamin A, SARS-CoV-2, network pharmacology, target, mechanism

## Abstract

Severe acute respiratory syndrome coronavirus 2 (SARS-CoV-2) causes coronavirus disease 2019 (COVID-19), an epidemic disease characterized by rapid infection and a high death toll. The clinical diagnosis of patients with COVID-19 has risen sharply, especially in Western countries. Globally, an effective treatment for COVID-19 is still limited. Vitamin A (VA) exhibits pharmacological activity in the management of pneumonia. Thus, we reason that VA may potentially serve as an anti-SARS-CoV-2 regimen. In this study, bioinformatics analysis and computation assays using a network pharmacology method were conducted to explore and uncover the therapeutic targets and mechanisms of VA for treating COVID-19. We identified candidate targets, pharmacological functions, and therapeutic pathways of VA against SARS-CoV-2. Bioinformatics findings indicate that the mechanisms of action of VA against SARS-CoV-2 include enrichment of immunoreaction, inhibition of inflammatory reaction, and biological processes related to reactive oxygen species. Furthermore, seven core targets of VA against COVID-19, including *MAPK1, IL10, EGFR, ICAM1, MAPK14, CAT*, and *PRKCB* were identified. With this bioinformatics-based report, we reveal, for the first time, the anti-SARS-CoV-2 functions and mechanisms of VA and suggest that VA may act as a potent treatment option for COVID-19, a deadly global epidemic.

## INTRODUCTION

Coronavirus disease 2019 (COVID-19), a deadly disease induced by the novel coronavirus severe acute respiratory syndrome coronavirus 2 (SARS-CoV-2), was first discovered in China, and is accompanied by a high degree of morbidity and mortality [1]. The virus has spread across the world and infected very large populations, especially in the United States [2]. Clinical features of COVID-19 may include pyrexia, asthenia, dyspnea, as well as acute respiratory distress syndrome, septic shock, and coagulation dysfunction in critically ill patients [3]. It is believed that, due to its high fatality rate, SARS-CoV-2 may strongly bind to angiotensin-converting enzyme 2 (ACE2), which is a key factor in the pathological pathway of SARS-CoV infection in the host [4]. The latest research findings have indicated that the affinity of SARS-CoV-2 to ACE2 may be 10 to 20 times greater than that of SARS-CoV [5]. In the SARS-CoV-2-infected population, it has been reported that people aged over 65 years have the highest rate of death [6]. Clinically, an effective and accurate diagnosis of SARS-CoV-2 has been achieved. However, the medical pharmacotherapy to treat COVID-19 is still insufficient [7]. Accordingly, it is necessary to explore and develop bioactive compounds to treat COVID-19. Vitamins are considered as a food supplement and have been reported to play an important role in the immune system [8]. Vitamin A (VA) has potent physiological functions, such as promoting growth and reproduction and maintaining bone, epithelial tissue, vision, and normal secretion of mucosal epithelium. VA and its derivatives can prevent precancerous lesions [9]. An increasing number of reports indicate that VA is necessary to maintain immune function and that it is responsible for immune cell differentiation and proliferation [10]. It has been reported that VA can improve the ability of immune cells to produce antibodies and induce T lymphocytes to release functional lymphokines through modulation of target genes via nuclear receptors [11]. Additionally, VA is extremely important for maintaining sufficient levels of natural “killer cells” (showing antiviral activity) in circulating blood [12]. VA adjuvant therapy may enhance body immune function by increasing IgM and IgG levels and activating T lymphocytes [13]. More importantly, vitamin A was reported to play a significant role against pneumonia. It is evidenced that low VA content is linked to neonatal pneumonia [14]. Clinical data show that VA deficiency is implicated in fatal mycoplasma-induced pneumonia in children [15]. In addition, VA supplementation contributes to the reduction of clinical complications and shortening of in-hospital time for children with pneumonia [16]. All these pieces of evidence suggest that vitamin A may be an optional treatment for COVID-19; however, to date there has been no investigation of VA against SARS-CoV-2, especially its pharmacological mechanism. In this report, we aimed to determine and identify the curative effect of VA for treating COVID-19 and to utilize a network pharmacology approach to uncover the mechanisms underlying the therapeutic role of VA.

## RESULTS

### Identification of SARS-CoV-2- and vitamin A-associated genes

**Figure 1:**
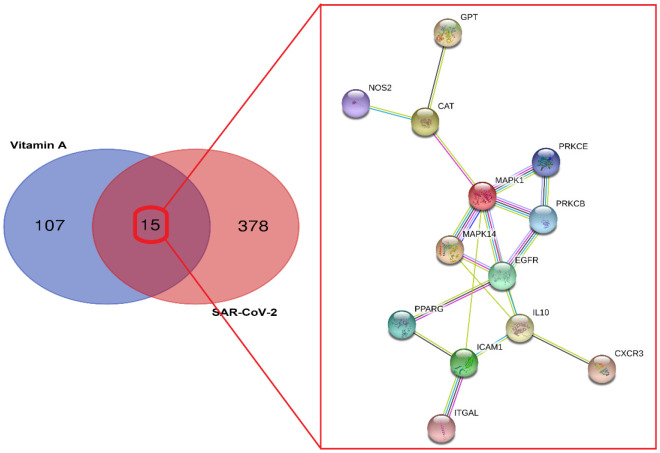
**Identification of vitamin A and SARS-CoV-2 associated genes.** Venn diagram showing the intersection targets of vitamin A against SARS-CoV-2 with an identified PPI network.

**Figure 2:**
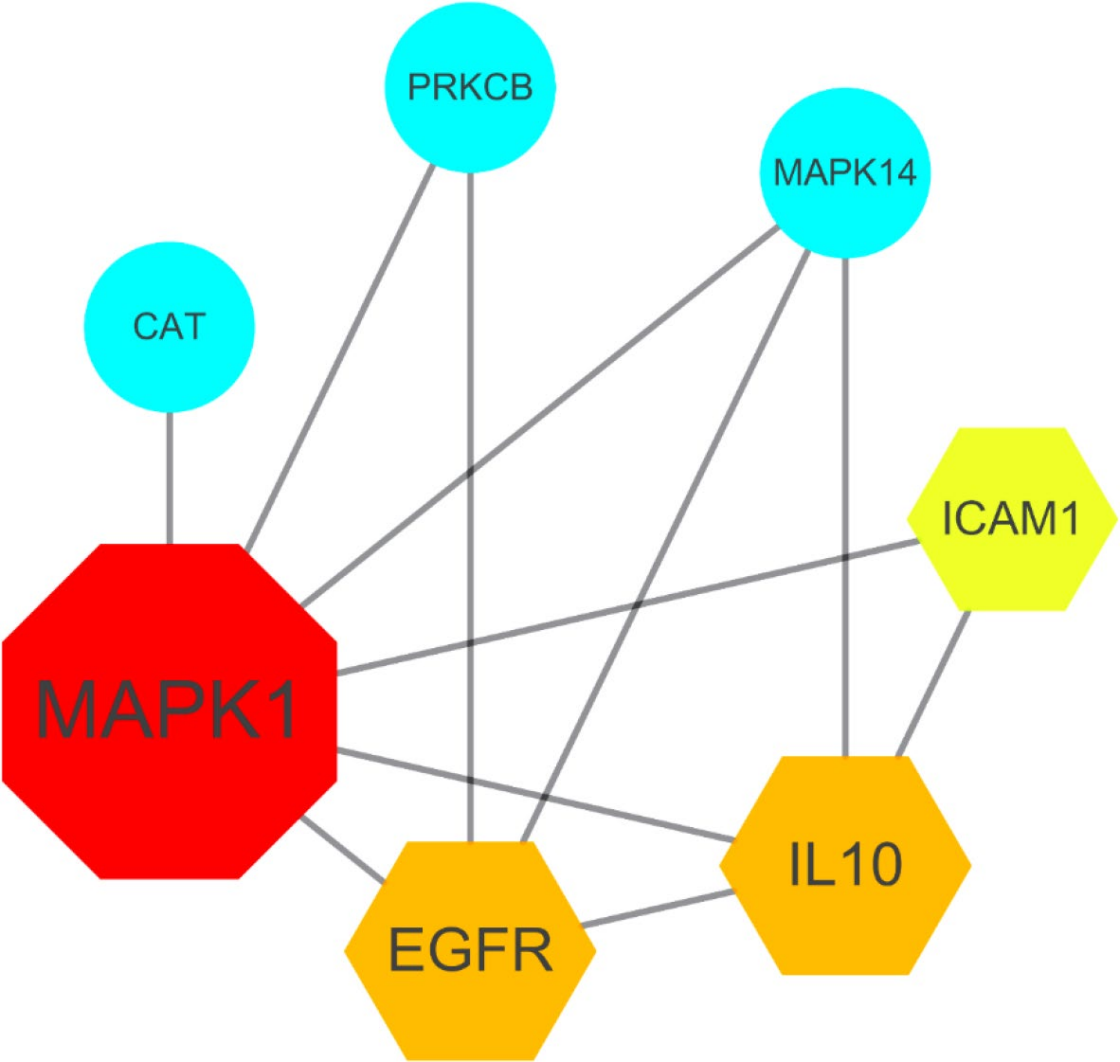
**Gene network of the seven core targets of vitamin A against SARS-CoV-2**

In order to determine the SARS-CoV-2-associated and VA-pharmacological action genes, we conducted a series of bioinformatic analyses. We assayed and identified 393 SARS-CoV-2-associated genes from the Genecard and OMIM datasets. In addition, 122 VA-pharmacological action genes were identified following data correction using the UniProt tool (Figure 1). When we compared the target (VA-associated and SARS-CoV-2-associated) genes, 15 VA-associated targets against SARS-CoV-2 were identified, and the common genes were subjected to target-function-protein interaction network analysis (Figure 1). By setting the median degree of freedom to 2.923, the maximum degree of freedom to 7, and the core target screening condition ranged to 3–7), seven core targets of VA against SARS-CoV-2 were identified, namely MAPK1, IL10, EGFR, ICAM1, MAPK14, CAT, and PRKCB (Figure 2).

### Biological role of vitamin A against SARS-CoV-2

**Figure 3:**
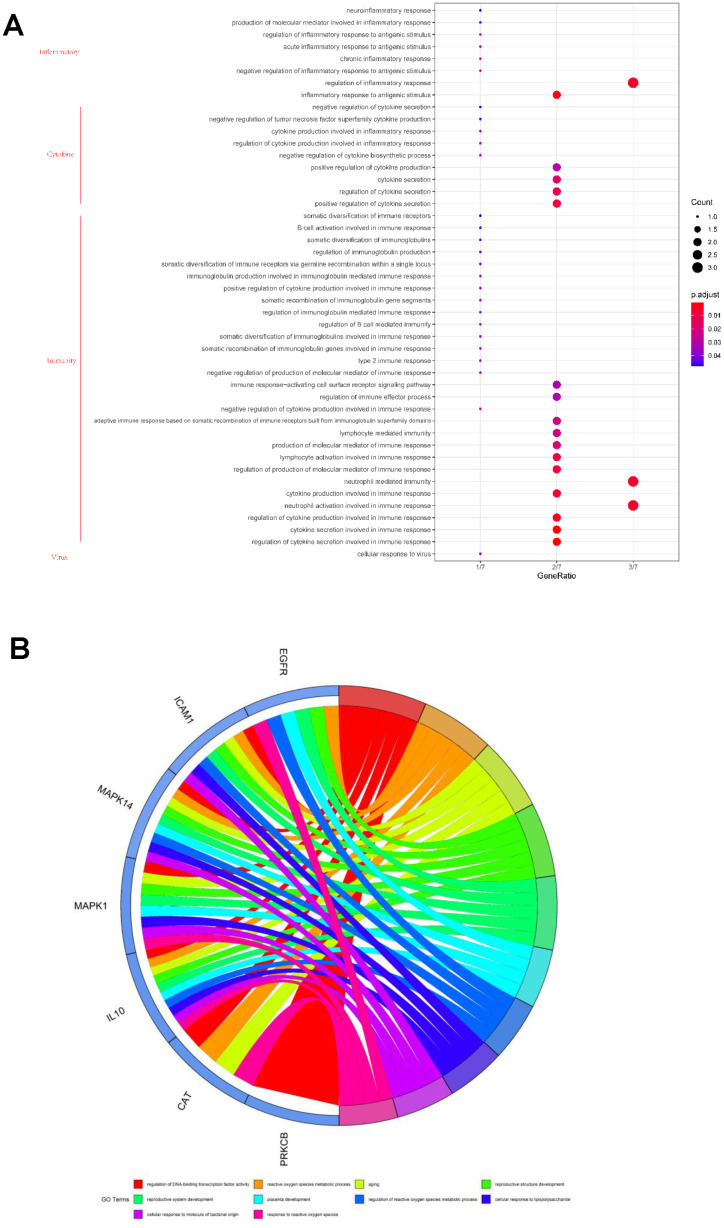
**Gene ontology analysis of the seven core targets of vitamin A against SARS-CoV-2.** (**A**) Bubble diagram showing the vitamin A-mediated biological process against SARS-CoV-2. (**B**) All core biotargets of vitamin A against SARS-CoV-2 were linked to the top 10 most enriched GO terms in Circro diagrams.

The core targets were subjected to gene ontology (GO) biological process and KEGG pathway enrichment analysis to understand the possible biological role of VA and the signaling pathway against SARS-CoV-2. The GO results highlighted that VA mediated a number of biological processes related to cellular response to the virus, immunity, cytokine production and secretion, and inflammatory response (Figure 3A and Supplementary Table 1). Immune responses are mediated by different classes of immune cells, such as neutrophils and lymphocytes, through the immune response-activating cell surface receptor signaling pathway. The VA-mediated immune response is also regulated by immunoglobulin production (Figure 3A and Supplementary Table 1). In addition, VA played a role in both acute and chronic inflammatory responses against SARS-CoV-2 (Figure 3A and Supplementary Table 1).

### Vitamin A-mediated signaling pathway and network against SARS-CoV-2

**Figure 4:**
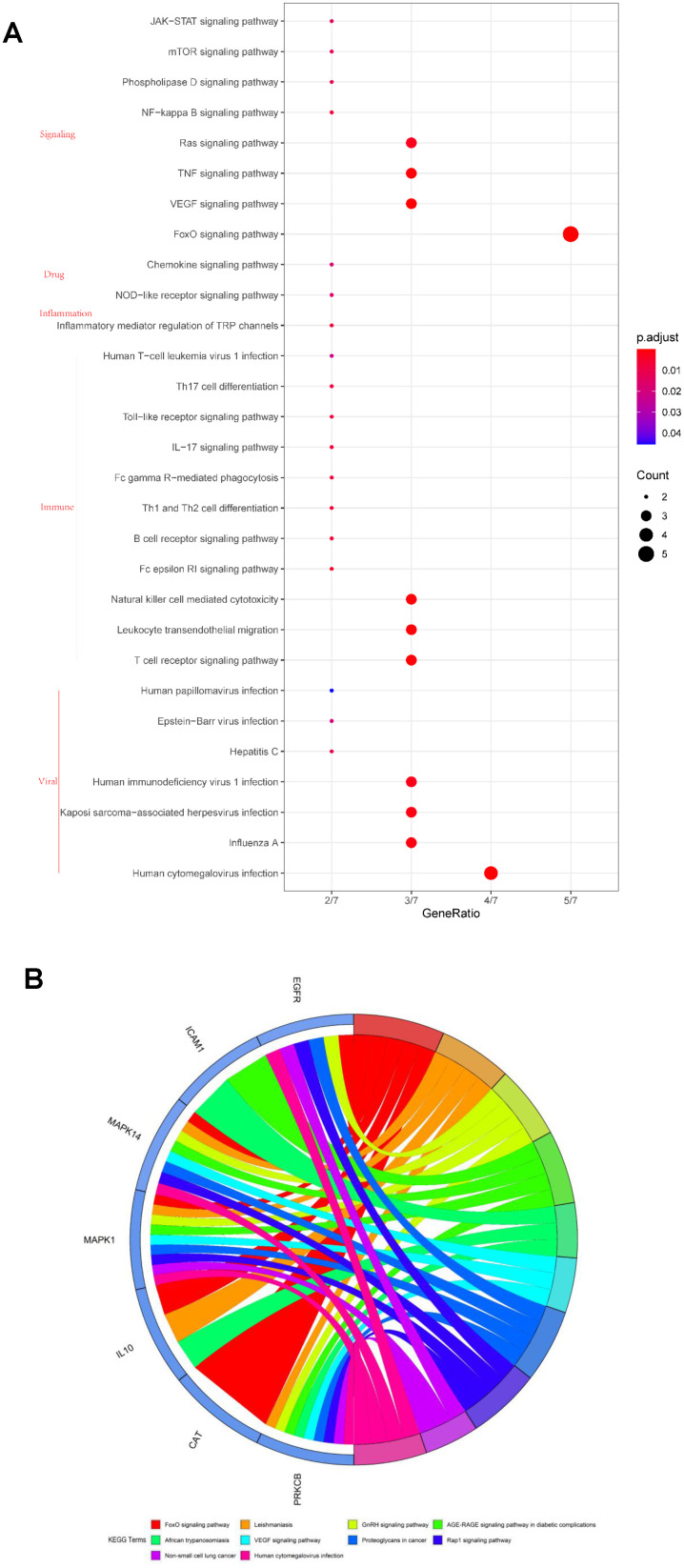
**Kyoto Encyclopedia of Genes and Genomes (KEGG) pathway analysis of the seven core targets of vitamin A against SARS-CoV-2.** (**A**) Bubble diagram showing the vitamin A-mediated cell signaling pathways against SARS-CoV-2. (**B**) Identified core biotargets of vitamin A against SARS-CoV-2 were associated with the 10 most enriched KEGG terms in Circro diagrams.

**Figure 5:**
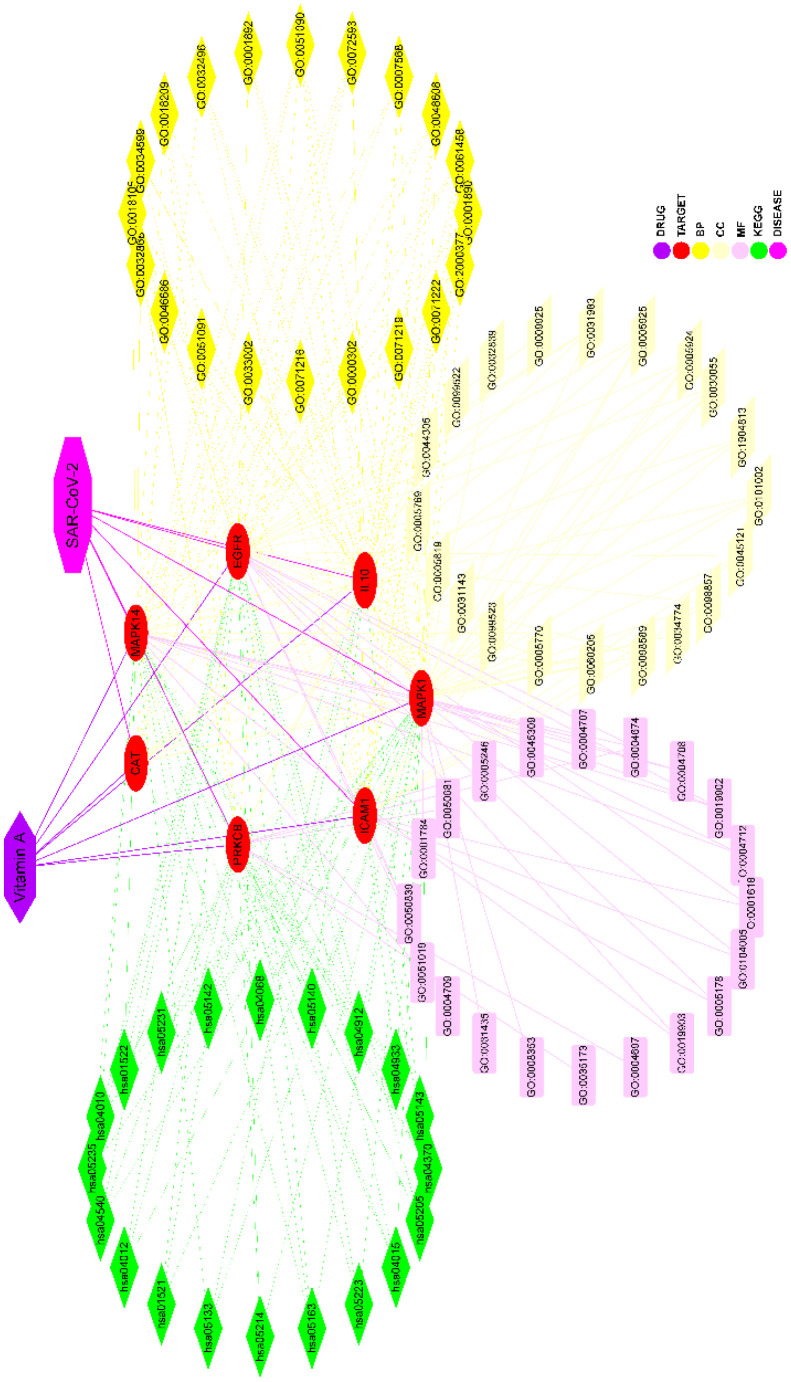
**Interaction network of the target-KEGG pathways of VA against SARS-CoV-2.** The middle part represented the anti-SARS-CoV-2 targets of vitamin A, and the enriched top 20 biological functions and KEGG pathways.

We conducted a KEGG pathway analysis to further understand the possible mechanism underlying the anti-SARS-CoV-2 role of vitamin A. Our results highlighted that vitamin A might regulate a series of signaling pathways related to viral infections, such as human cytomegalovirus, influenza A, Kaposi sarcoma-associated herpes virus, human immunodeficiency virus 1, hepatitis C, Epstein-Barr, and human papilloma virus (Figure 4 and Online Resource 2). In addition, we also found that vitamin A was involved in many important cell signaling pathways such as FoxO, VEGF, TNF, Ras, nuclear factor kappa B (NF-κB), phospholipase D, mTOR, and JAK-STAT (Figure 4 and Supplementary Table 2). More importantly, we found the involvement of vitamin A in immune responses such as T cell receptor signaling pathway, leukocyte transendothelial migration, natural killer cell mediated cytotoxicity, Fc epsilon RI signaling pathway, B cell receptor signaling pathway, Th1 and Th2 cell differentiation, Fc gamma R-mediated phagocytosis, IL-17 signaling pathway, Toll-like receptor signaling pathway, Th17 cell differentiation, and human T-cell leukemia virus 1 infection (Figure 4 and Supplementary Table 2). Vitamin A regulates cytokine production via NOD-like receptor signaling pathway and chemokine signaling pathway (Figure 4 and Supplementary Table 2). These findings suggest that vitamin A could be a potential treatment for COVID-19. Finally, we used Cytoscape software to construct the gene network diagram of VA-target-GO-KEGG-SARS-CoV-2 (Figure 5).

## DISCUSSION

Recently, the prevalence and mortality of SARS-CoV-2 has increased sharply worldwide, especially in developed countries in 2020 [17]. However, there are few existing drug treatments for COVID-19, and some of the immunotherapy treatments and antiviral drugs used in clinical practice have shown limited therapeutic effectiveness [18]. Thus, further investigation and development of potential therapeutic agents is warranted. In this bioinformatics report, we aimed to identify and reveal the anti-SARS-CoV-2 targets and molecular mechanisms of VA through an emerging approach to network pharmacology. VA may exert potent, beneficial pharmacological activity for the treatment of COVID-19 via associated cytoprotection, anti-viral and anti-inflammatory effects, and immunoregulation. By using the network pharmacology strategy to uncover the molecular functions, the anti-SARS-CoV-2 effects of VA could be shown to be modulated by some key molecules and corresponding genes, including *MAPK1*, *IL10*, *EGFR*, *ICAM1*, *MAPK14*, *CAT*, and *PRKCB*. In our previous report, we demonstrated that vitamin C could also modulate a cluster of core targets against SARS-CoV-2 [19]. When we compared the findings, we found that two core targets, MAPK1 and EGFR, were found in the effect of both vitamin A and vitamin C. MAPK1, a functional protein kinase, is a key connection in the switch from extracellular irritation to intracellular signaling. Changes in the signaling pathway are evidenced in complex diseases, including cancers [20]. It has been reported that anti-pneumonia action exerted by VA is related to specific suppression of the MAPK signaling pathway, including MAPK1 activity [21]. EGFR, a tyrosine kinase receptor, plays important roles in modulating cell proliferation, division, differentiation, survival, and oncogenesis [22]. It has been reported that EGFR-mutated patients are likely to have hospital-acquired pneumonia [23]. IL-10, a pivotal anti-inflammatory cytokine, can effectively control inflammatory Th cells and immunopathology and secure cellular homeostasis [24]. Some reports have shown that inactivation of IL-10 is linked to an increased risk of developing pneumonia [25–26]. ICAM-1, a transmembrane glycoprotein receptor, can recruit inflammatory cells and cytokines to target tissue [27]. Reportedly, blood ICAM-1 content may function as a potent biomarker of patients with pneumonia [28], including pediatric pneumonia [29]. Catalase, a well-known antioxidant enzyme, acts as an oxidative catalyst for some biological functions [30]. Biologically, catalase is found to have potential therapeutic effectiveness against influenza-induced pneumonia [31]. PRKCB, an important regulator of B cells, can regulate metabolic and mitochondrial reprogramming responsible for B cell fate [32]. Some evidence indicates that PRKCB overexpression is associated with the development of pneumonia via activation of the NF-κB pathway [33]. Collectively, these predictive peptides may be used as potential markers for detecting SARS-CoV-2 and may serve as pharmacological targets against SARS-CoV. In further bioinformatics and computational assays, the therapeutic mechanisms of VA for managing COVID-19 could be conjunctively actualized through collective regulation of the FoxO signaling pathway, GnRH signaling pathway, PD-L1 expression, and PD-1 checkpoint pathway. Accordingly, VA demonstrates several pharmacological mechanisms against SARS-CoV, namely, cytoprotective action, anti-viral activity, anti-inflammatory effects, and immunity-based immunomodulation. The anti-coronavirus benefits may be the dual efficacy of a nutrient agent and bioactive compound to treat complex disease by synergistically modulating all presumptive multi-targets and multi-pathways. Adjuvant supplementation of VA may enhance the therapeutic efficacy of current clinical anti-viral agents and immunotherapy to treat potentially fatal COVID-19. However, the current findings should be further validated clinically.

**Figure 6:**
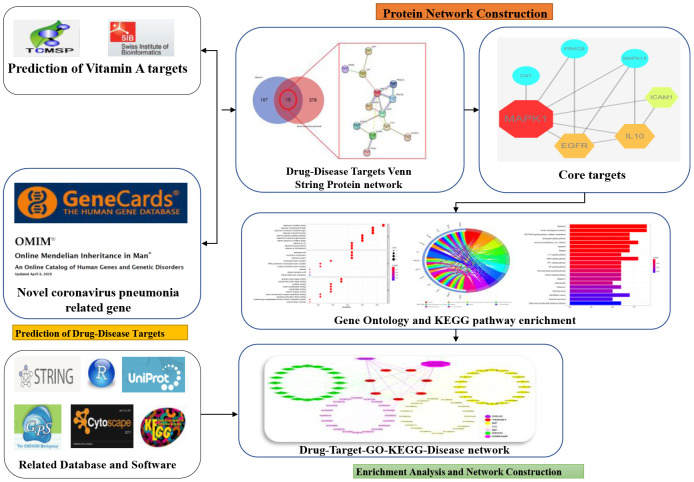
**A schematic diagram to summarize the workflow of the study.**

In conclusion, the bioinformatics and computational findings from this study highlight the role of vitamin A in anti-viral, anti-inflammatory, and immunomodulatory effects via different biological processes and cell signaling pathways, as revealed by network pharmacology analysis. More importantly, VA may be used clinically for the treatment of COVID-19, as evidenced by the identified biological processes—which indicate pharmacological functions—and the signaling pathways, which suggest therapeutic mechanisms (Figure 6).

## MATERIALS AND METHODS

### Identification of candidate genes activated by VA

By using online datasets, in particular TCMSP, Drugbank, SuperPred, Swiss Target Prediction, ChemMapper, and BATMAN TCM, all target genes activated by VA were obtained. The genes activated by VA were collected for target correction from reviewed (Swiss-Prot) and Uniprot databases. The Genecard and OMIM datasets were employed to harvest SARS-CoV-2 genes. All the shortlisted genes and targets of VA and SARS-CoV-2 were subjected to intersection analysis via an online platform with graphical output (http://bioinformatics.psb.ugent.be/webtools/Venn/) in the form of Venn diagrams were plotted to display the relational VA-activated genes and targets of VA against SARS-CoV-2 [34, 35].

### Gene Ontology (GO) and KEGG pathway enrichment analyses

The *R*-language package ClusterProfiler, ReactomePA, org.Hs.eg.Db, and GOplot were applied in the assay and visualization of the relational targets. GO analysis was conducted with the use of org.Hs.eg.Db, with a p-value cutoff of 0.05 in enriched output of bubble charts and Circos-circle charts. The Pathview package in *R*-language was used to merge the relational targets of enriched KEGG pathways by drawing pathway diagrams [36, 37].

### Construction of network visualization in core targets

The software Cytoscape (v3.7.1) was used to plot the component-target-pathway network and the GO biological process and KEGG pathway-based visual graphics of VA against SARS-CoV-2 [38, 39].

### Establishment of the biological process and KEGG molecular pathway of VA

To compare the anti-SARS-CoV-2 effects of VA, bioinformatics data of top biological functions and KEGG-enriched pathways were applied for pairwise comparison, including the value of -log10 (p-adjust) as the heat-map parameter, and the heat-map was drawn using HemI 1.0 software [19, 40].

### Construction of construction of protein-protein interaction (PPI) network graphics of core targets

The relational targets of VA against SARS-CoV were employed as inputs to the software STRING for plotting target-to-target network interactions and target interaction PPI network diagram. The NetworkAnalyzer setting in Cytoscape software was applied to analyze topology parameters, such as median degrees of freedom and maximum degrees of freedom in the network. The optimal targets were collected based on degree values (DV). The upper limit of the filtering range was the maximum DV in the topology data, and the lower limit was twice the median of freedom, as previously reported [41, 42].

## Supplementary Material

Supplementary Table 1

Supplementary Table 2

## References

[1] Rubin EJ, Baden LR, Morrissey S. Audio interview: lessons from covid-19 hotspots. N Engl J Med. 2020; 382:e35. 10.1056/NEJMe2007783 32242382

[2] Kaplan EH. Containing 2019-nCoV (Wuhan) coronavirus. Health Care Manag Sci. 2020; 23:311–14. 10.1007/s10729-020-09504-6 32146554 PMC7087552

[3] Chu DK, Pan Y, Cheng SM, Hui KP, Krishnan P, Liu Y, Ng DY, Wan CK, Yang P, Wang Q, Peiris M, Poon LL. Molecular diagnosis of a novel coronavirus (2019-nCoV) causing an outbreak of pneumonia. Clin Chem. 2020; 66:549–55. 10.1093/clinchem/hvaa029 32031583 PMC7108203

[4] Li W, Moore MJ, Vasilieva N, Sui J, Wong SK, Berne MA, Somasundaran M, Sullivan JL, Luzuriaga K, Greenough TC, Choe H, Farzan M. Angiotensin-converting enzyme 2 is a functional receptor for the SARS coronavirus. Nature. 2003; 426:450–54. 10.1038/nature02145 14647384 PMC7095016

[5] Cao Y, Li L, Feng Z, Wan S, Huang P, Sun X, Wen F, Huang X, Ning G, Wang W. Comparative genetic analysis of the novel coronavirus (2019-nCoV/SARS-CoV-2) receptor ACE2 in different populations. Cell Discov. 2020; 6:11. 10.1038/s41421-020-0147-1 32133153 PMC7040011

[6] Zhang G, Zhang J, Wang B, Zhu X, Wang Q, Qiu S. Analysis of clinical characteristics and laboratory findings of 95 cases of 2019 novel coronavirus pneumonia in Wuhan, China: a retrospective analysis. Respir Res. 2020; 21:74. 10.1186/s12931-020-01338-8 32216803 PMC7099829

[7] Lu H. Drug treatment options for the 2019-new coronavirus (2019-nCoV). Biosci Trends. 2020; 14:69–71. 10.5582/bst.2020.01020 31996494

[8] Spinas E, Saggini A, Kritas SK, Cerulli G, Caraffa A, Antinolfi P, Pantalone A, Frydas A, Tei M, Speziali A, Saggini R, Pandolfi F, Conti P. Can vitamin a mediate immunity and inflammation? J Biol Regul Homeost Agents. 2015; 29:1–6. 25864736

[9] Soares MM, Silva MA, Garcia PP, Silva LS, Costa GD, Araújo RM, Cotta RM. Efect of vitamin a suplementation: a systematic review. Cien Saude Colet. 2019; 24:827–38. 10.1590/1413-81232018243.07112017 30892504

[10] Ang A, Pullar JM, Currie MJ, Vissers MC. Vitamin C and immune cell function in inflammation and cancer. Biochem Soc Trans. 2018; 46:1147–59. 10.1042/BST20180169 30301842 PMC6195639

[11] Stephensen CB. Vitamin A, infection, and immune function. Annu Rev Nutr. 2001; 21:167–92. 10.1146/annurev.nutr.21.1.167 11375434

[12] Lee H, Ko G. New perspectives regarding the antiviral effect of vitamin a on norovirus using modulation of gut microbiota. Gut Microbes. 2017; 8:616–20. 10.1080/19490976.2017.1353842 28727498 PMC5730389

[13] Zhang P, Cui TT, Zhang ZH, Wang YQ. Low-dose vitamin a therapy on T lymphocyte function in neonatal pneumonia. Eur Rev Med Pharmacol Sci. 2018; 22:4371–74. 10.26355/eurrev_201807_15437 30024627

[14] Tian Y, Tian Q, Wu Y, Peng X, Chen Y, Li Q, Zhang G, Tian X, Ren L, Luo Z. Vitamin a supplement after neonatal streptococcus pneumoniae pneumonia inhibits the progression of experimental asthma by altering CD4^+^T cell subsets. Sci Rep. 2020; 10:4214. 10.1038/s41598-020-60665-4 32144294 PMC7060180

[15] Xing Y, Sheng K, Xiao X, Li J, Wei H, Liu L, Zhou W, Tong X. Vitamin a deficiency is associated with severe Mycoplasma pneumoniae pneumonia in children. Ann Transl Med. 2020; 8:120. 10.21037/atm.2020.02.33 32175413 PMC7049042

[16] Hu N, Li QB, Zou SY. [Effect of vitamin a as an adjuvant therapy for pneumonia in children: a meta analysis]. Zhongguo Dang Dai Er Ke Za Zhi. 2018; 20:146–53. 29429465 10.7499/j.issn.1008-8830.2018.02.013 PMC7389232

[17] Ji D, Juhas M, Tsang CM, Kwok CK, Li Y, Zhang Y. Discovery of g-quadruplex-forming sequences in SARS-CoV-2. Brief Bioinform. 2020. [Epub ahead of print]. 10.1093/bib/bbaa114 32484220 PMC7314185

[18] AminJafari A, Ghasemi S. The possible of immunotherapy for COVID-19: a systematic review. Int Immunopharmacol. 2020; 83:106455. 10.1016/j.intimp.2020.106455 32272396 PMC7128194

[19] Li R, Wu K, Li Y, Liang X, Lai KP, Chen J. Integrative pharmacological mechanism of vitamin C combined with glycyrrhizic acid against COVID-19: findings of bioinformatics analyses. Brief Bioinform. 2020. [Epub ahead of print]. 10.1093/bib/bbaa141 32662814 PMC7462346

[20] Braicu C, Buse M, Busuioc C, Drula R, Gulei D, Raduly L, Rusu A, Irimie A, Atanasov AG, Slaby O, Ionescu C, Berindan-Neagoe I. A comprehensive review on MAPK: a promising therapeutic target in cancer. Cancers (Basel). 2019; 11:1618. 10.3390/cancers11101618 31652660 PMC6827047

[21] Dai J, Gu L, Su Y, Wang Q, Zhao Y, Chen X, Deng H, Li W, Wang G, Li K. Inhibition of curcumin on influenza a virus infection and influenzal pneumonia via oxidative stress, TLR2/4, p38/JNK MAPK and NF-κB pathways. Int Immunopharmacol. 2018; 54:177–87. 10.1016/j.intimp.2017.11.009 29153953

[22] Sabbah DA, Hajjo R, Sweidan K. Review on epidermal growth factor receptor (EGFR) structure, signaling pathways, interactions, and recent updates of EGFR inhibitors. Curr Top Med Chem. 2020; 20:815–34. 10.2174/1568026620666200303123102 32124699

[23] Choi WI, Jeong J, Lee CW. Association between EGFR mutation and ageing, history of pneumonia and gastroesophageal reflux disease among patients with advanced lung cancer. Eur J Cancer. 2019; 122:101–08. 10.1016/j.ejca.2019.09.010 31634646

[24] Fang D, Zhu J. Molecular switches for regulating the differentiation of inflammatory and IL-10-producing anti-inflammatory t-helper cells. Cell Mol Life Sci. 2020; 77:289–303. 10.1007/s00018-019-03277-0 31432236 PMC11105075

[25] Ding S, Wang X, Chen W, Fang Y, Liu B, Liu Y, Fei G, Wang L. Decreased interleukin-10 responses in children with severe mycoplasma pneumoniae pneumonia. PLoS One. 2016; 11:e0146397. 10.1371/journal.pone.0146397 26751073 PMC4708986

[26] Chakraborty K, Raundhal M, Chen BB, Morse C, Tyurina YY, Khare A, Oriss TB, Huff R, Lee JS, St Croix CM, Watkins S, Mallampalli RK, Kagan VE, et al. The mito-DAMP cardiolipin blocks IL-10 production causing persistent inflammation during bacterial pneumonia. Nat Commun. 2017; 8:13944. 10.1038/ncomms13944 28074841 PMC5241690

[27] Mukhopadhyay S, Malik P, Arora SK, Mukherjee TK. Intercellular adhesion molecule-1 as a drug target in asthma and rhinitis. Respirology. 2014; 19:508–13. 10.1111/resp.12285 24689994

[28] Abo-Hagar HH, Abo-Elezz AA, Mehrez M, Mabrouk MM, Elshora OA. Diagnostic efficacy of serum amyloid a protein and soluble intercellular adhesion molecule 1 in pediatric ventilator-associated pneumonia. J Intensive Care Med. 2019; 34:503–10. 10.1177/0885066617702598 28403662

[29] Chang PY, Tsao SM, Chang JH, Chien MH, Hung WY, Huang YW, Yang SF. Plasma levels of soluble intercellular adhesion molecule-1 as a biomarker for disease severity of patients with community-acquired pneumonia. Clin Chim Acta. 2016; 463:174–80. 10.1016/j.cca.2016.10.030 27983998

[30] Glorieux C, Calderon PB. Catalase, a remarkable enzyme: targeting the oldest antioxidant enzyme to find a new cancer treatment approach. Biol Chem. 2017; 398:1095–108. 10.1515/hsz-2017-0131 28384098

[31] Shi X, Shi Z, Huang H, Zhu H, Zhu H, Ju D, Zhou P. PEGylated human catalase elicits potent therapeutic effects on H1N1 influenza-induced pneumonia in mice. Appl Microbiol Biotechnol. 2013; 97:10025–33. 10.1007/s00253-013-4775-3 23525936 PMC7079947

[32] Tsui C, Martinez-Martin N, Gaya M, Maldonado P, Llorian M, Legrave NM, Rossi M, MacRae JI, Cameron AJ, Parker PJ, Leitges M, Bruckbauer A, Batista FD. Protein kinase c-β dictates B cell fate by regulating mitochondrial remodeling, metabolic reprogramming, and heme biosynthesis. Immunity. 2018; 48:1144–59.e5. 10.1016/j.immuni.2018.04.031 29884460 PMC6015119

[33] Vielma SA, Krings G, Lopes-Virella MF. Chlamydophila pneumoniae induces ICAM-1 expression in human aortic endothelial cells via protein kinase c-dependent activation of nuclear factor-kappaB. Circ Res. 2003; 92:1130–37. 10.1161/01.RES.0000074001.46892.1C 12714566

[34] Su M, Guo C, Liu M, Liang X, Yang B. Therapeutic targets of vitamin C on liver injury and associated biological mechanisms: a study of network pharmacology. Int Immunopharmacol. 2019; 66:383–87. 10.1016/j.intimp.2018.11.048 30530052

[35] Wu K, Wei P, Liu M, Liang X, Su M. To reveal pharmacological targets and molecular mechanisms of curcumol against interstitial cystitis. J Adv Res. 2019; 20:43–50. 10.1016/j.jare.2019.05.003 31193808 PMC6543129

[36] Zhou R, Wu K, Su M, Li R. Bioinformatic and experimental data decipher the pharmacological targets and mechanisms of plumbagin against hepatocellular carcinoma. Environ Toxicol Pharmacol. 2019; 70:103200. 10.1016/j.etap.2019.103200 31158732

[37] Li R, Guo C, Li Y, Qin Z, Huang W. Therapeutic targets and signaling mechanisms of vitamin C activity against sepsis: a bioinformatics study. Brief Bioinform. 2020. [Epub ahead of print]. 10.1093/bib/bbaa079 32393985 PMC7454291

[38] Li R, Guo C, Li Y, Liang X, Yang L, Huang W. Therapeutic target and molecular mechanism of vitamin c-treated pneumonia: a systematic study of network pharmacology. Food Funct. 2020; 11:4765–72. 10.1039/d0fo00421a 32420559

[39] Li R, Song Y, Ji Z, Li L, Zhou L. Pharmacological biotargets and the molecular mechanisms of oxyresveratrol treating colorectal cancer: network and experimental analyses. Biofactors. 2020; 46:158–67. 10.1002/biof.1583 31647596

[40] Li R, Ma X, Song Y, Zhang Y, Xiong W, Li L, Zhou L. Anti-colorectal cancer targets of resveratrol and biological molecular mechanism: analyses of network pharmacology, human and experimental data. J Cell Biochem. 2019. [Epub ahead of print]. 10.1002/jcb.28404 30719773

[41] Ge B, Guo C, Liang Y, Liu M, Wu K. Network analysis, and human and animal studies disclose the anticystitis glandularis effects of vitamin C. Biofactors. 2019; 45:912–19. 10.1002/biof.1558 31469455

[42] Liang Y, Zhou R, Liang X, Kong X, Yang B. Pharmacological targets and molecular mechanisms of plumbagin to treat colorectal cancer: a systematic pharmacology study. Eur J Pharmacol. 2020; 881:173227. 10.1016/j.ejphar.2020.173227 32505664

