# Supplementary material for: Revealing the targets and mechanisms of vitamin A in the treatment of COVID-19
Source: Aging (Albany NY). 2020 Aug 15;12(15):15784–96. doi: 10.18632/aging.103888 (PMC7467385; doi:10.18632/aging.103888)
Supplement: Supplementary Table 2 [file aging-12-103888-s002..pdf]

SUPPLEMENTARY TABLE

Supplementary Table 2. KEGG pathway enrichment analysis using the identified seven core targets of VA against SARS-CoV-2.

ID	Description	GeneRatio	BgRatio	pvalue	p.adjust	qvalue	geneID	Count	remark
hsa05163	Human cytomegalovirus infection	4/7	225/8025	1.97E-05	0.000313	8.71E-05	MAPK1/EGFR/MAPK14/PRKCB	4	viral
hsa05164	Influenza A	3/7	170/8025	0.000307	0.00132	0.000367	MAPK1/ICAM1/PRKCB	3	viral
hsa05167	Kaposi sarcoma-associated herpesvirus infection	3/7	186/8025	0.0004	0.001632	0.000454	MAPK1/ICAM1/MAPK14	3	viral
hsa05170	Human immunodeficiency virus 1 infection	3/7	212/8025	0.000588	0.002175	0.000605	MAPK1/MAPK14/PRKCB	3	viral
hsa05160	Hepatitis C	2/7	155/8025	0.007304	0.012354	0.003435	MAPK1/EGFR	2	viral
hsa05169	Epstein-Barr virus infection	2/7	201/8025	0.012066	0.019176	0.005332	ICAM1/MAPK14	2	viral
hsa05165	Human papillomavirus infection	2/7	330/8025	0.030868	0.045444	0.012636	MAPK1/EGFR	2	viral
hsa04660	T cell receptor signaling pathway	3/7	104/8025	7.13E-05	0.000515	0.000143	MAPK1/IL10/MAPK14	3	immune
hsa04670	Leukocyte transendothelial migration	3/7	112/8025	8.89E-05	0.000524	0.000146	ICAM1/MAPK14/PRKCB	3	immune
hsa04650	Natural killer cell mediated cytotoxicity	3/7	131/8025	0.000142	0.000705	0.000196	MAPK1/ICAM1/PRKCB	3	immune
hsa04664	Fc epsilon RI signaling pathway	2/7	68/8025	0.001446	0.00442	0.001229	MAPK1/MAPK14	2	immune
hsa04662	B cell receptor signaling pathway	2/7	82/8025	0.002095	0.005552	0.001544	MAPK1/PRKCB	2	immune
hsa04658	Th1 and Th2 cell differentiation	2/7	92/8025	0.00263	0.006744	0.001875	MAPK1/MAPK14	2	immune
hsa04666	Fc gamma R-mediated phagocytosis	2/7	93/8025	0.002687	0.00678	0.001885	MAPK1/PRKCB	2	immune
hsa04657	IL-17 signaling pathway	2/7	94/8025	0.002744	0.006817	0.001895	MAPK1/MAPK14	2	immune
hsa04620	Toll-like receptor signaling pathway	2/7	104/8025	0.003348	0.007293	0.002028	MAPK1/MAPK14	2	immune
hsa04659	Th17 cell differentiation	2/7	107/8025	0.003541	0.007608	0.002115	MAPK1/MAPK14	2	immune
hsa05166	Human T-cell leukemia virus 1 infection	2/7	219/8025	0.014222	0.021743	0.006046	MAPK1/ICAM1	2	immune
hsa04750	Inflammatory mediator regulation of TRP channels	2/7	100/8025	0.0031	0.007142	0.001986	MAPK14/PRKCB	2	inflammation
hsa04621	NOD-like receptor signaling pathway	2/7	181/8025	0.009861	0.016164	0.004494	MAPK1/MAPK14	2	drug

hsa04062	Chemokine signaling pathway	2/7	189/8025	0.010718	0.01739	0.004835	MAPK1/PRKCB	2	drug
hsa04068	FoxO signaling pathway	5/7	131/8025	2.20E-08	3.49E-06	9.71E-07	MAPK1/IL10/EGFR/MAPK14/CAT	5	signaling
hsa04370	VEGF signaling pathway	3/7	59/8025	1.29E-05	0.000298	8.29E-05	MAPK1/MAPK14/PRKCB	3	signaling
hsa04668	TNF signaling pathway	3/7	112/8025	8.89E-05	0.000524	0.000146	MAPK1/ICAM1/MAPK14	3	signaling
hsa04014	Ras signaling pathway	3/7	232/8025	0.000766	0.002767	0.000769	MAPK1/EGFR/PRKCB	3	signaling
hsa04064	NF-kappa B signaling pathway	2/7	104/8025	0.003348	0.007293	0.002028	ICAM1/PRKCB	2	signaling
hsa04072	Phospholipase D signaling pathway	2/7	148/8025	0.006676	0.011819	0.003286	MAPK1/EGFR	2	signaling
hsa04150	mTOR signaling pathway	2/7	155/8025	0.007304	0.012354	0.003435	MAPK1/PRKCB	2	signaling
hsa04630	JAK-STAT signaling pathway	2/7	162/8025	0.007957	0.013179	0.003664	IL10/EGFR	2	signaling
